# SARS-CoV-2 Prevalence on and Incidence after Arrival in Travelers on Direct Flights from Cape Town, South Africa to Munich, Germany Shortly after Occurrence of the Omicron Variant in November/December 2021: Results from the OMTRAIR Study

**DOI:** 10.3390/pathogens12020354

**Published:** 2023-02-20

**Authors:** Cornelia Seidl, Liza Coyer, Nikolaus Ackermann, Katharina Katz, Jan Walter, Siegfried Ippisch, Martin Hoch, Merle M. Böhmer

**Affiliations:** 1 Infectious Disease Epidemiology and Surveillance Unit, Bavarian Health and Food Safety Authority, 80636 Munich, Germany; 2 Postgraduate Training in Applied Epidemiology (PAE), Department of Infectious Epidemiology, Robert Koch Institute, 13353 Berlin, Germany; 3 ECDC Fellowship Programme, Field Epidemiology path (EPIET), European Centre for Disease Prevention and Control, 16 973 Solna, Sweden; 4 Public Health Microbiology Unit, Bavarian Health and Food Safety Authority, 85764 Oberschleissheim, Germany; 5 Task Force Infectious Diseases Department, Bavarian Health and Food Safety Authority, 80636 Munich, Germany; 6 Institute of Social Medicine and Health Systems Research, Otto-von-Guericke-University, 39120 Magdeburg, Germany

**Keywords:** COVID-19, SARS-CoV-2, coronavirus, variant, Omicron, aircraft, air travel, preventive measures, transmission

## Abstract

The highly transmissible SARS-CoV-2-variant B.1.1.529 (Omicron) first appeared in South Africa in November 2021. In order to study Omicron entry to Germany, its occurrence related to incoming airline travel, symptomatology and compliance with entry regulations and recommendations, we conducted a cross-sectional study, followed by a retrospective cohort study among passengers and crew on 19 direct flights from Cape Town, South Africa, to Munich, Germany, between 26 November and 23 December 2021. Travelers were mandatorily PCR-tested on arrival and invited to complete an online questionnaire. SARS-CoV-2-prevalence on arrival was 3.3% (n = 90/2728), and 93% were Omicron. Of the passengers, 528 (19%) completed the questionnaire. Among participants who tested negative on arrival, self-reported SARS-CoV-2-incidence was 4.3% within 14 days, of whom 74% reported a negative PCR-test ≤ 48 h before boarding, 77% were fully vaccinated, and 90% reported wearing an FFP2/medical mask during flight. We found multiple associations between risk factors and infection on and after arrival, among which having a positive-tested travel partner was the most noteworthy. In conclusion, PCR testing before departure was insufficient to control the introduction of the Omicron variant. Additional measures (e.g., frequent testing, quarantine after arrival or travel ban) should be considered to delay virus introduction in such settings.

## 1. Introduction

The highly transmissible SARS-CoV-2-variant B.1.1.529 (Omicron) was first detected in South Africa on 9 November 2021 [1,2]. Quickly thereafter, Omicron became the dominant SARS-CoV-2 variant worldwide [3,4], likely as the result of its high transmissibility, although a high rate of international air travel probably has contributed too [5,6,7].

There have been multiple reports of flight-associated SARS-CoV-2 transmission [8,9,10,11]. The risk of in-flight transmission is generally considered low due to the usage of HEPA filters, vertical airflow and seats acting as physical barriers [12], but infections could still be imported and could spread at travelers’ destinations if left unnoticed and undetected. In order to prevent flight-associated SARS-CoV-2 importation and transmission, pre-departure polymerase chain reaction (PCR) testing, non-pharmaceutical interventions such as masking and physical distancing before, during and after the flight, and quarantine are commonly implemented measures among countries with COVID pandemic protective measures. However, little data are available about the effectiveness of these methods, and the true extent to which SARS-CoV-2 infections are imported and transmitted through air travel is unclear [13]. In addition, most studies assessing the occurrence of SARS-CoV-2 infections related to airline travel to date were conducted before the introduction of vaccination and non-pharmaceutical interventions and before the emergence of Omicron [7,8,14,15].

Omicron was first detected in the German federal state of Bavaria (~13.1 million inhabitants) in a couple who traveled by plane from South Africa to Munich on 24 November 2021 and who self-initiated variant-specific diagnostics following initial media reports on Omicron [16]. To limit the risks of further Omicron importation and transmission, the German government implemented a series of control measures, including mandatory testing before leaving or entering the country, quarantine for travelers from Variant of Concern (VOC) risk areas, the obligation to fill in a digital registration on entry and wearing of facemasks during air travel. In addition, the Bavarian Ministry of Health implemented mandatory PCR testing for all crewmembers and passengers aged six years or older arriving by direct flight from Cape Town, South Africa, at Munich Airport, between 26 November and 23 December 2021. Passengers and crew were also required to fill in a Passenger Locator Card (PLC) and provide further information on contact details, flight date and seat number. This information was forwarded to the local public health authority for the purpose of health monitoring during the first 14 days after arrival and for international contact tracing.

To study Omicron entry to Germany, its occurrence among airline travelers and on-board staff on arrival and shortly after, symptomatology and compliance with entry regulations and recommendations, we conducted a cross-sectional study on the prevalence of SARS-CoV-2 using PCR testing on arrival data, followed by a retrospective cohort study on self-reported incident infections, among passengers and crew on 19 direct flights from Cape Town, South Africa, to Munich, Germany, between 26 November and 23 December 2021. These data will allow us to inform future flight-related measures to prevent the importation and spread of new VOCs.

## 2. Materials and Methods

### 2.1. Study Design and Population

We conducted a cross-sectional study, followed by a retrospective cohort study, among all crew and passengers aged six years and older who traveled by direct flight from Cape Town, South Africa, to Munich, Germany, between 26 November 2021 and 23 December 2021. Excluded were travelers exempt from the mandatory testing obligation, which included non-Schengen travelers who continued their journey to another destination, and, from 15 December 2021, passengers with a final destination in another European Union (EU) country and a valid, negative PCR test before departure in South Africa.

### 2.2. Procedures

All travelers who arrived on the above-mentioned flights received a mandatory oropharyngeal swab upon arrival at Munich Airport. From 26 November to 12 December 2021, rapid PCR (PoC-PCR) testing was used, and from 13 December 2021 onward, a standard PCR procedure was implemented to reduce the rate of required retesting at the airport. Passengers with positive PCR tests at Munich Airport entered an isolation period of 14 days and were followed up by confirmatory PCR testing at the laboratory of the Bavarian Health and Food Safety Authority (LGL), Oberschleissheim, Germany. Positive confirmatory PCR tests were followed by further diagnostics (i.e., variant-specific PCR and genome sequencing for Omicron). According to the Bavarian Omicron control measures, all passengers and staff were required to complete a PLC including contact details, to begin a 14-day quarantine after arrival, and to immediately notify the local public authority and test for SARS-CoV-2 if they showed COVID-19 symptoms during their quarantine period. The obligation to complete a digital registration form before arrival and to enter quarantine after arrival did not apply to crews who stayed less than 72 h in a VOC area.

Between 18 February and 15 June 2022, we sent an email to travelers based on the information provided in their PLC. We invited them to complete an anonymous questionnaire within the OMicron TRansmission in AIRcraft (OMTRAIR)-study on the online platform LamaPoll. Travelers who tested positive for SARS-CoV-2 at the airport but whose result could not be confirmed by confirmatory PCR were not invited. We contacted all invited travelers with available phone contact details individually by phone to ask whether they had received the questionnaire and to remind them to complete it. There were two versions of the questionnaire: (1) one for travelers who tested negative on arrival and (2) one for travelers with a confirmed positive test on arrival. Both versions were available in German and English and accessible via computer, tablet and mobile phone. The ethics committee of the Bavarian State Medical Association (Bayerische Landesärztekammer) declared that the OMTRAIR study does not require ethics approval because it was considered part of the quality assurance process of public health measures. We obtained digital informed consent from all study participants at the start of the questionnaire. For travelers aged 6–13 years, we asked their parents or legal guardians to complete the questionnaire for them. Adolescents aged 14–17 years could complete the questionnaire independently or with the help of their parents/guardians.

### 2.3. Primary Outcomes

SARS-CoV-2 status upon arrival for all travelers was based on the PCR test result at the airport and the confirmatory PCR test at the LGL laboratory. From 26 November to 12 December 2021, oropharyngeal swab specimens were tested for SARS-CoV-2 using a rapid PCR test (Bio-Speedy SARS-CoV-2 Double Gene RT-qPCR; Bioeksen R&D Technologies, County Cork, Ireland) [17]. From 13 December 2021, a standard PCR procedure was implemented using ampliCube Coronavirus SARS-CoV-2 (Mikrogen Diagnostik, Neuried, Germany) [18]. At the LGL laboratory, the SARS-CoV-2 variant was determined using TIB Molbiol VirSNiP SARS-CoV-2 assays (TIB Molbiol, Berlin, Germany) [19]. For confirmation, genome sequencing using the Illumina COVIDSeq test (Illumina, San Diego, CA, USA) was performed [19,20]. SARS-CoV-2 status in the 14 days after arrival (i.e., incident infection) was based on self-reported questionnaire data.

### 2.4. Information from Questionnaire

We collected the following information from study participants based on self-report in the study questionnaire: demographics (i.e., month and year of birth, gender), flight information (i.e., date of arrival and seat on the aircraft), information on entry regulations (i.e., SARS-CoV-2 testing prior to flight, quarantine), chronic conditions, COVID-19 vaccination status, history of SARS-CoV-2 infection prior to flight, travel details and behaviors in South Africa (i.e., wearing of mask and type of mask, hand washing, hand disinfection, distance to other persons, transportation type, staying in crowds, contact with a person who tested positive, and travel partner tested positive), and behaviors during flight (i.e., wearing of mask and type of mask, contact with other people, and activities during flight). Additionally, we asked participants who tested negative on arrival if they tested positive for SARS-CoV-2 within 14 days after arrival, and if yes, the date of the positive test and the type of test used. We asked participants who tested positive on or within 14 days after arrival for information on the SARS-CoV-2 variant and on symptomatology, date of onset, and duration of illness.

We defined compliance with German entry regulations as fulfilling the following three requirements: (1) proof of negative SARS-CoV-2 test before boarding (antigen test of maximum 24 h old or nucleic acid test, e.g., PCR test, of maximum 72 h old) for persons aged 12 years or older; (2) completed a digital entry registration form before entering Germany; and (3) remained in quarantine after arrival (or in isolation if tested positive) regardless of vaccination or recovery status.

### 2.5. Statistical Analysis

We calculated SARS-CoV-2 prevalence with a 95% confidence interval (CI) on arrival by dividing the number of confirmed infections by the number of all tested passengers and crew, excluding non-confirmed infections.

We categorized participants of the questionnaire into four groups: (1) positive on arrival, (2) negative on arrival but positive within 14 days after arrival, (3) negative on arrival and did not become positive within 14 days after arrival, and (4) negative on arrival and missing information about infection status in the 14 days after arrival. We described demographic characteristics and compliance with entry regulations and prevention measures before arrival, overall, and stratified by SARS-CoV-2 infection status. We calculated incidence within 14 days after arrival with 95% CI by dividing the number of participants who reported testing positive by the total number of participants who were PCR negative on arrival and who had information on incident infection.

**Figure A1:**
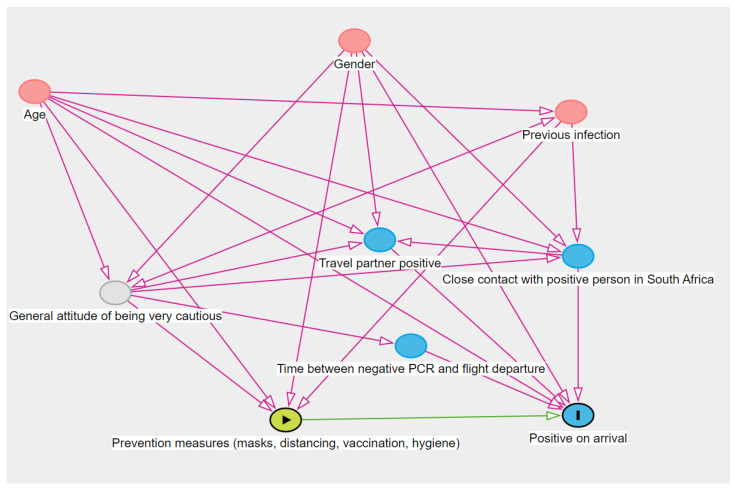
Directed acyclic graph of characteristics, entry regulations, prevention measures and behaviors in South Africa associated with testing positive on arrival.

**Figure A2:**
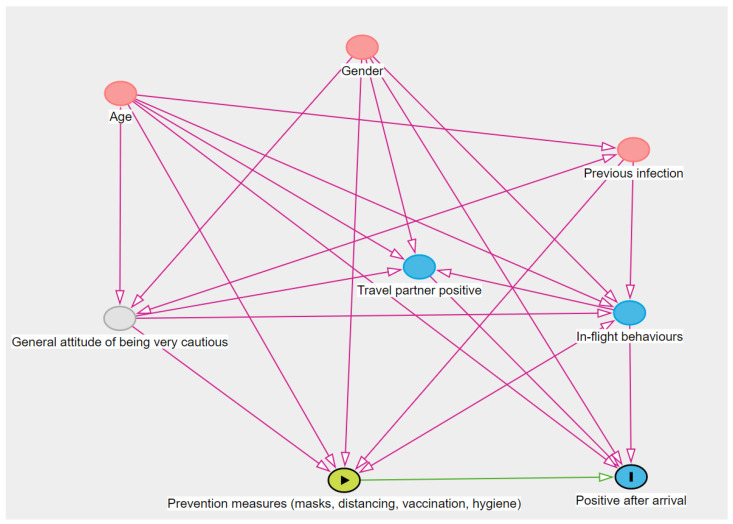
Directed acyclic graph of characteristics, prevention measures and in-flight behaviors associated with testing positive within 14 days after arrival (i.e., incident infection).

We created a directed acyclic graph (DAG) representing hypothesized causal associations with testing positive on arrival (including characteristics, entry regulations, preventions measures and behaviors in South Africa) and incident infection within the 14 days after arrival among those negative on arrival (including characteristics, prevention measures and in-flight behaviors) (i.e., two DAGs, shown in Appendix A Figure A1 and Figure A2). Using log-binomial regression, we evaluated univariable associations of each factor with the respective outcome. For each association, we calculated relative risk ratios (RR) and 95% confidence intervals (95% CIs). For each statistically significant association, we then identified potential confounders from the DAG. As we were unable to build a final multivariable model for each exposure-outcome association due to sparse data, we added each potential confounder to the model one by one to assess confounding (i.e., whether the association would change in terms of statistical significance or ≥10% change in the RR).

A *p*-value < 0.05 was considered statistically significant. All data management, analysis, and visualization were conducted using R version 4.0.2 (Vienna, Austria).

## 3. Results

### 3.1. SARS-CoV-2 Prevalence on Arrival

**Figure 1:**
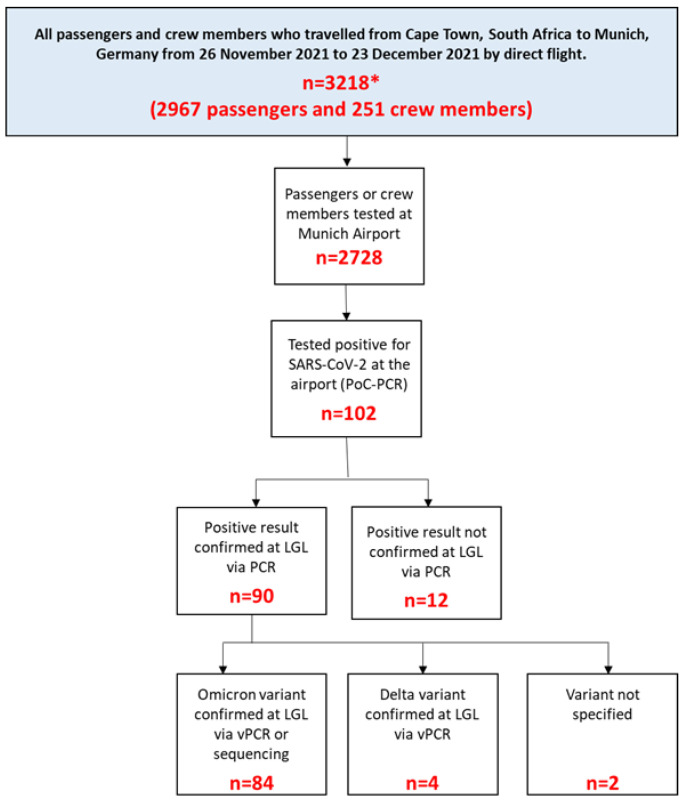
Flowchart of tests performed on passengers and crew who traveled by direct flight from Cape Town, South Africa, to Munich, Germany, during the period 26 November–23 December 2021. PCR = polymerase chain reaction; LGL = Bavarian Health and Food Safety Authority; vPCR = variant-specific PCR; * total including those passengers from non-Schengen countries, children aged < 6 years (no testing obligation), from 15 December 2021 onwards also passengers with final destination in another EU country who had a PCR test that was not older than 48 h.

From 26 November through 23 December 2021, a total of 2728 passengers and crew on 19 direct flights from Cape Town, South Africa, to Munich, Germany, underwent PCR testing at Munich Airport. Of 2728 tested travelers, 102 tested positive at the airport, of which 90 were confirmed at the LGL laboratory (Figure 1). As such, SARS-CoV-2 prevalence on arrival was 3.3% (n = 90/2728, 95%CI = 2.6–4.0%). Sixteen out of 19 flights had positive travelers on arrival (Appendix A Table A1). Most (84/90, 93%) travelers who were confirmed positive had the Omicron variant.

### 3.2. SARS-CoV-2 Incidence within 14 Days after Arrival among Study Participants

**Figure 2:**
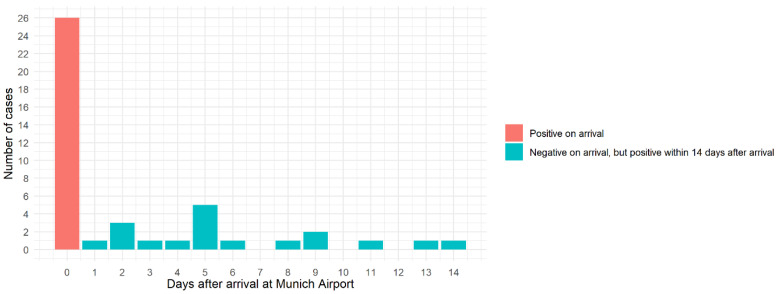
Time of positive test result relative to arrival at Munich Airport in days among participants who tested positive on (n = 26) or within 14 days after (n = 21) arrival and completed the OMTRAIR study questionnaire, November/December 2021 n = 3 missing from the figure.

Of 2728 passengers with a SARS-CoV-2 test result on arrival, 528 (19%) completed the study questionnaire. The median age of participants was 49 years (interquartile range: 36–60); 245 (46%) identified as female; 452 (92%) were passengers; and 26 (4.9%) tested positive on arrival (Table 1). Among the 490 travelers with a negative test on arrival and information on testing within 14 days after arrival, 21 (4.3%, 95%CI = 2.5–6.0%) reported testing positive within 14 days after arrival (n = 11 and n = 4 after 5 and 10 days, respectively), 16 (76%) of which had Omicron. All infected individuals were passengers; none were crew. Figure 2 shows the time of positive test results relative to arrival at Munich Airport in days.

### 3.3. Symptoms

Of all 37 participants with Omicron on (n = 21) or within 14 days after (n = 16) arrival who filled in the study questionnaire, 28 (78%) reported clinical symptoms (Appendix A Table A2). The most common symptom was headache (n = 27/32, 84%), followed by fatigue (n = 25/32, 78%), cough (n = 21/32, 66%), sore throat (n = 18/32, 56%) and rhinitis (n = 17/32, 53%). Two patients reported a decrease or loss of smell or taste. There were no reports of hospital admissions or death.

### 3.4. Compliance with Entry Regulations and Prevention Measures

Table 2 summarizes compliance with entry regulation and prevention measures before arrival at Munich Airport, overall and by SARS-CoV-2 infection status among participants of the study questionnaire. After 28 November 2021, an obligation to furnish proof of a negative SARS-CoV-2 test result before departure applied to all travelers entering Germany. Twenty-nine reported not being in possession of a negative test result before departure, and four did not know, even though it was mandatory at this point in time and should be checked by the carrier before boarding. Of the passengers, 389 (74%) reported a negative PCR test not older than 48 h before boarding (73% and 81% among those positive on and within 14 days after arrival, respectively). A total of 405 (77%) were fully vaccinated (85% and 86% among those positive on and within 14 days after arrival, respectively).

### 3.5. Associations with Testing Positive on Arrival

Reporting a positive travel partner, always or often wearing masks indoors and outdoors or avoiding mass transportation (vs. never or rarely) and sometimes avoiding contact with people outside the travel group (vs. never or rarely) were associated with an increased risk of testing positive on arrival in univariable analysis (Table 3). All associations remained statistically significant in the models adjusting for confounders, except for avoiding contact with people outside the travel group (Appendix A Table A3).

### 3.6. Associations with Incident Infection

Female gender, age ≤ 34 years, and reporting a positive travel partner were associated with an increased risk of incident infection (Table 4). Gender and reporting a positive travel partner remained statistically significant in the models adjusting for confounders (Appendix A Table A4).

## 4. Discussion

In this study, we investigated SARS-CoV-2 occurrence among passengers and crew on 19 direct flights from Cape Town, South Africa, to Munich, Germany, between 26 November and 23 December 2021. We tested all travelers on arrival at Munich Airport and asked them to complete a questionnaire on travel details, entry regulations and prevention measures, pre-, during, and post-flight behaviors, and incident infection within 14 days after arrival. We found a 3.3% prevalence of SARS-CoV-2 among all travelers on arrival and a 4.3% self-reported incidence of SARS-CoV-2 among questionnaire participants. Some 75% of participants complied with entry regulations and infection prevention measures. Travelers with a SARS-CoV-2-positive travel partner were more likely to test positive both on and after arrival. Always or often wearing masks indoors and outdoors and avoiding mass transportation were associated with an increased risk of testing positive on arrival. Female travelers were more likely to have an incident infection.

Omicron infections accounted for 76% of prevalent and incident SARS-CoV-2 infections in our study. Reports from other countries indeed indicate that Omicron was imported to Europe between late November and December 2021 and quickly became the dominant variant [3,4,21,22]. In line with other studies indicating lower severity of infection with the Omicron-BA.1 variant compared to infections with the Delta variant [23,24,25], most Omicron infections in our study had mild symptomology. In addition, a decrease or loss of smell or taste was relatively rare [26]. Reduced severity is probably related to lower virulence of Omicron as well as infection-acquired immunity and higher vaccination coverage in the population compared to previous infection waves [24,25]. Indeed, in our study, 77% of participants were fully vaccinated, with similar vaccination coverage among participants with and without infection, which also reflects reduced effectiveness in preventing infection of prevailing vaccinations [25]. In addition, travelers might generally be in relatively good health.

Despite mandatory pre-departure testing, our study found a SARS-CoV-2 prevalence on arrival of 3.3%. These findings indicate that mandatory pre-departure testing did not detect all infections, as could be expected, given the time lag between testing and departure [26,27,28] and suboptimal test accuracy [29,30,31,32]. Thus, additional measures are needed to identify undetected infections and prevent further transmission, such as testing on arrival, as was conducted in our study. Unfortunately, implementation of mandatory screening at borders is very resource-intensive, might not be logistically feasible, and might not be applicable or feasible for certain subpopulations (i.e., young children and transit passengers). However, it might be possible to implement and actively promote voluntary on-arrival testing. In Germany, many airports provide testing, although it is not free of charge. Mandatory test regimes might be reserved for a very early stage of entry of a VOC into a hitherto unaffected area. Another possible approach to contain the spread of new variants in the early stages might be imposing temporary travel restrictions on non-essential travel, as far as possible, considering a ban on the carriage of travelers does not apply to German citizens and persons who have a right of residence in the Federal Republic of Germany [33] In this respect, the situation in November/December in Bavaria was very favorable in that a new VOC was spreading from a single country with only a few easily controllable flight connections. This may not be the case with the emergence of future VOCs.

We found that 4.3% of travelers who initially tested negative on arrival reported testing positive in the 14 days after arrival. However, it is difficult to determine whether these travelers acquired their infection inflight, given that the majority tested positive within 5 days after the flight. In general, the determination of the onset of infection in asymptomatic persons by testing remains challenging. The overall risk of during-flight transmission is considered very low [13]. On the other hand, there are multiple case series reporting possible in-flight transmission of SARS-CoV-2 [8,9,10,11]. We also cannot completely rule out SARS-CoV-2 acquisition prior to departure, especially among those with a positive travel partner. However, the vast majority of participants complied with entry regulations, including pre-boarding testing and vaccination, and none reported contact with a positive person in South Africa. We assume post-flight transmission to be relatively low as well, considering the high compliance with quarantine. Furthermore, the majority of passengers with incident infections reported infection with Omicron. At the time, Omicron was not a dominant variant in Europe, with only a few reported cases outside of South Africa and surrounding countries by late November and December 2021 [25].

Travel-related infections that are not detected by pre-boarding or on-arrival testing can be imported and spread at the traveler´s destination. Quarantine [27,28,34,35] and increased testing shortly after arrival [36,37], followed by isolation if positive, could be effective strategies to prevent or delay transmission depending on the length of quarantine and uptake of testing. In our study, compliance with mandatory self-isolation and quarantine for 14 days was high at 100% and 80%, respectively. Additionally, the majority of participants who tested negative on arrival accessed further testing during quarantine, suggesting an easy and high availability of tests at their destination in Germany.

We identified several factors putatively associated with prevalent and incident infection. Similar to our findings, another study also found that a positive travel partner is associated with a higher risk of incident infection [9]. Female travelers may have been more likely to have a positive travel partner. We also observed some counterintuitive associations between prevention measures and infection, which warrant further study, particularly because variation in responses was limited for some measures. These associations could have potentially been introduced by unmeasured or residual confounding or might be explained by selection bias if people who unexpectedly acquired an infection despite applying prevention measures were more likely to participate in our study than people who did not.

Strengths of this study include the full testing coverage on arrival, in line with the mandatory policy by the Bavarian government, the timing of the study at the start of the Omicron wave with a single “country of origin” connected by only a few flights and no significant other travel options, and the ability of following-up passengers after arrival to determine incidence. However, our study is not without limitations. First, the response rate of the questionnaire was low at 19%, despite email and telephone reminders. The difficulty of contacting and following up with airline travelers is a known challenge [8]. In our case, it could be explained by incomplete or missing information on the PLCs, as these were not checked for plausibility. Related to this, second, the relatively low number of participants, particularly with an infection on or after arrival, may have led to sparse data. We were unable to evaluate certain relationships of factors with infection, as there were too few participants in some of the strata. We were also unable to conduct a full multivariable analysis, although our assessments of confounding suggest it was likely limited. In addition, the power to detect differences between groups based on demographic characteristics and prevention measures was low. Third, there may be selection bias. Travelers who tested positive on or after arrival might have been more motivated to participate in our study than travelers who did not. This might have led to an overestimation of the incidence rate. Moreover, severely ill, hospitalized or deceased patients were likely not willing nor able to participate in our study, hence clinical symptoms and outcomes might have been underreported. Fourth, all measures except for the test result on arrival were self-reported. This could have led to social desirability bias or recall bias if persons who became ill after the flight remembered events or behaved differently than passengers who did not become ill. Fifth, passengers poorly reported information on seat numbers, and we could not compare Next Generation Sequencing (NGS)-results of the individual passengers for data protection reasons. Therefore, we could not investigate proximity to a case during flight as a potential risk factor nor establish whether transmission occurred in-flight or around flight because infections that occurred within 14 days after a negative test on arrival might have been acquired prior to or after traveling. However, we assume post-flight transmission to be relatively low, considering the high compliance with quarantine in the study sample.

## 5. Conclusions

In conclusion, despite entry regulations and prevention measures, a relatively high proportion of travelers tested positive for SARS-CoV-2 on or shortly after arrival in Munich. Whereas some transmissions might have occurred during or around the flight, PCR test results of asymptomatic persons, in general, must be considered momentary snapshots. Therefore, our study demonstrates that if PCR testing were performed only once before departure or upon arrival, some cases would be missed. Measures such as multiple PCR tests in the days after the flight as well as quarantine, could be considered to prevent (or at least delay) SARS-CoV-2 importation and transmission, particularly of new and potentially harmful variants in unaffected areas.

## Figures and Tables

**Table 1:** Characteristics of passengers and crew traveling from Cape Town, South Africa, to Munich, Germany, in November/December 2021 and included in the OMTRAIR study.

Characteristics	Total	SARS-CoV-2 Test Result on or within 14 Days after Arrival at Munich Airport			
		Positive on Arrival	Negative on Arrival but Positive within 14 Days after Arrival	Negative on Arrival and Did Not Become Positive within 14 Days after Arrival	Negative on Arrival and No Information on Infection within 14 Days after Arrival ^†^
	N = 528 (100%)	n = 26 (4.9%)	n = 21 (4.2%)	n = 469 (89%)	n = 12 (2.3%)
**Role during flight**					
Passenger	452 (92%)	25 (100%)	20 (100%)	405 (91%)	2 (100%)
Crew	41 (8.3%)	0 (0%)	0 (0%)	41 (9.2%)	0 (0%)
**Age in years (median [IQR])**					
	49 [36–60]				
6–18	8 (1.6%)	1 (3.8%)	1 (4.8%)	6 (1.3%)	0 (0%)
19–34	113 (22%)	14 (54%)	13 (62%)	231 (49%)	1 (33%)
35–64	318 (62%)	9 (35%)	6 (29%)	156 (33%)	1 (33%)
≥65	78 (15%)	2 (7.7%)	1 (4.8%)	74 (16%)	1 (33%)
**Gender**					
Male	272 (53%)	14 (54%)	5 (24%)	251 (54%)	2 (67%)
Female	245 (47%)	12 (46%)	16 (76%)	216 (46%)	1 (33%)
Diverse	1 (0.2%)	0 (0%)	0 (0%)	1 (0.2%)	0 (0%)
**SARS-CoV-2 variant, for those testing positive on or within 14 days after arrival (n = 47)**					
Omicron	37 (80%)	21 (81%)	16 (80%)	n.a.	n.a.
Delta	3 (6.5%)	1 (3.8%)	2 (10%)	n.a.	n.a.
Unknown	6 (13%)	4 (15%)	2 (10%)	n.a.	n.a.
**Any other travelers in the travel group tested positive for SARS-CoV-2**					
Yes	60 (13%)	7 (30%)	9 (45%)	44 (10%)	0 (0%)
No	321 (67%)	11 (48%)	8 (40%)	301 (70%)	1 (100%)
Unknown	95 (20%)	5 (22%)	3 (15%)	87 (20%)	0 (0%)
**Close contact in South Africa with a person who subsequently tested positive for SARS-CoV-2**					
Yes	72 (15%)	5 (21%)	3 (15%)	64 (15%)	0 (0%)
No	318 (66%)	15 (62%)	15 (75%)	288 (66%)	0 (0%)
Unknown	92 (19%)	4 (17%)	2 (10%)	85 (19%)	1 (100%)
**Any underlying chronic condition**					
Yes	29 (15%)	7 (27%)	3 (14%)	3 (0.6%)	0 (0%)
No	433 (84%)	19 (73%)	18 (86%)	395 (85%)	1 (100%)
Unknown	3 (0.6%)	0 (0%)	0 (0%)	3 (0.6%)	0 (0%)

n.a. = not applicable. IQR = interquartile range; ^†^ Question on further testing after arrival was not answered. There were missing data for the role during flight (n = 35), age (n = 11), gender (n = 10), SARS-CoV-2 variant (n = 1), positive travel partner (n = 52), close contact in South Africa tested positive for SARS-CoV-2 (n = 46), underlying chronic condition (n = 13).

**Table 2:** Compliance with entry regulations and prevention measures among participants of the OMTRAIR study, Germany.

Regulation or Measure ^#^	Total	SARS-CoV-2 Test Result on or within 14 Days after Arrival at Munich Airport			
		Positive on Arrival	Negative on Arrival but Positive within 14 Days after Arrival	Negative on Arrival and Did Not Become Positive within 14 Days after Arrival	Negative on Arrival and No Information on Infection within 14 Days after Arrival Available
	N = 528 (100%)	n = 26 (4.9%)	n = 21 (4%)	n = 469 (89%)	n = 12 (2%)
**Vaccination status ^†^**					
Fully vaccinated	405 (77%)	22 (85%)	18 (86%)	364 (78%)	1 (8.3%)
Not fully vaccinated	62 (12%)	1 (3.8%)	3 (14%)	57 (12%)	1 (8.3%)
Not vaccinated	19 (3.6%)	1 (3.8%)	0 (0%)	18 (3.8%)	0 (0%)
**Type of mask worn during flight**					
FFP2 (KN95) mask or multiple mask types, including FFP2	383 (80%)	21 (88%)	18 (90%)	343 (79%)	1 (100%)
Only medical mask	91 (19%)	3 (12%)	2 (10%)	86 (20%)	0 (0%)
Fabric mask or no mask	7 (1.5%)	0 (0%)	0 (0%)	7 (1.6%)	0 (0%)
**Negative SARS-CoV-2 test result before departure**					
***Yes, PCR test***	392 (74%)	19 (86%)	17 (89%)	355 (84%)	1 (100%)
<12 h old	19 (4.1%)	0 (0%)	0 (0%)	19 (4.5%)	0 (0%)
≥12 to <24 h old	159 (34%)	6 (27%)	7 (37%)	146 (34%)	0 (0%)
≥24 to <48 h old	211 (45%)	13 (59%)	10 (53%)	187 (44%)	1 (100%)
Unknown period of time	3 (0.64%)	0 (0%)	0 (0%)	3 (0.7%)	0 (0%)
***Yes, antigen test***	22 (4.7%)	2 (9.1%)	1 (5.3%)	19 (4.5%)	0 (0%)
<12 h old	9 (2%)	0 (0%)	0 (0%)	9 (2.1%)	0 (0%)
≥12 to <24 h old	9 (2%)	2 (9.1%)	1 (5.3%)	6 (1.4%)	0 (0%)
≥24 to <48 h old	3 (0.64%)	0 (0%)	0 (0%)	3 (0.7%)	0 (0%)
Unknown period of time	1 (0.21%)	0 (0%)	0 (0%)	1 (0.24%)	0 (0%)
***Yes, but unknown type***	1 (0.2%)	0 (0%)	0 (0%)	1 (0.2%)	0 (0%)
***No test***	48 (10%)	1 (4.5%)	0 (0%)	47 (11%)	0 (0%)
***Unknown***	4 (0.9%)	0 (0%)	1 (5.3%)	3 (0.7%)	0 (0%)
**Quarantine/Isolation after arrival**					
Yes	393 (80%)	26 (100%)	21 (100%)	372 (80%)	1 (100%)
No	96 (20%)	0	0	95 (20%)	0 (0%)
**Fulfilled entry regulations ^§^**					
Yes	360 (76%)	21 (96%)	17 (94%)	321 (74%)	1 (100%)
No	116 (24%)	1 (4.5%)	1 (5.5%)	114 (26%)	0

PCR = polymerase chain reaction. The question on further testing after arrival was not answered. ^#^ There were missing data for vaccination status (n = 42), type of mask worn during flight (n = 47), SARS-CoV-2 test result before departure (n = 61), quarantine/isolation after arrival (n = 39) and entry regulations (n = 52). ^†^ Fully vaccinated was defined as 2 doses of COVID-19 vaccine Comirnaty/Spikevax/Vaxzevria or at least 1 dose of COVID-19 vaccine Janssen with the last shot given at least 14 days ago or recovered from SARS-CoV-2-infection and one dose of any vaccine approved in the EU. Proof of recovery was defined as a previous SARS-CoV-2-infection, which was confirmed by means of a nucleic acid test (e.g., PCR) and the test to prove the prior infection was performed no less than 28 days and no more than 90 days beforehand, according to the German Infection Protection Act. ^§^ Travelers who at any time within the last 10 days prior to entry have visited a VOC risk area must complete a digital entry registration before entering Germany, have to provide a negative test result (applies only to persons aged 12 years or older) and have to stay in quarantine for 14 days. This also applies to vaccinated and recovered individuals.

**Table 3:** Univariable associations of characteristics, entry regulations, prevention measures and behaviors in South Africa with testing positive at Munich Airport on arrival among those who filled in the OMTRAIR questionnaire.

	Total	Tested Positive for SARS-CoV-2 on Arrival at Munich Airport		Univariable Analysis	
Characteristics		Yes	No	RR (95% CI)	*p*-Value
	N = 528 (100%)	n = 26 (4.9%)	n = 502 (95%)		
**Age, years**					
>34	121 (23%)	7 (6%)	114 (94%)	Ref.	
≤35	396 (77%)	19 (5%)	377 (95%)	0.83 (0.37–2.08)	0.66
**Gender**					
Male	272 (53%)	14 (5%)	258 (95%)	Ref.	
Female	245 (47%)	12 (5%)	233 (95%)	0.95 (0.44–2.02)	0.90
Diverse	1 (0.2%)	0 (0%)	1 (100%)	Omitted	
**Vaccination status †**					
Not (fully) vaccinated	81 (17%)	2 (2.5%)	79 (97.5%)	Ref.	
Fully vaccinated	405 (83%)	22 (5%)	383 (95%)	2.20 (0.67–13.6)	0.28
**Previous SARS-CoV-2 infection**					
No/unknown	437 (87%)	22 (5%)	415 (95%)	Ref.	
Yes	65 (13%)	3 (5%)	62 (95%)	0.92 (0.22–2.55)	0.89
**Negative SARS-CoV-2 test result before departure**					
No/unknown	52 (11%)	1 (2%)	51 (98%)	Ref.	
Yes	415 (89%)	21 (5%)	349 (95%)	2.63 (0.57–46.7)	0.34
**Time between any negative test and departure**					
≥24 to <48 h	211 (46%)	13 (6.2%)	198 (94%)	Ref.	
< 24 h	199 (43%)	8 (4.0%)	191 (96%)	0.65 (0.26–1.51)	0.33
No test	48 (10%)	1 (2.1%)	47 (98%)	Omitted	
**Travel partner tested positive for SARS-CoV-2**					
No/unknown	416 (87%)	16 (4%)	400 (96%)	Ref.	
Yes	60 (13%)	7 (12%)	53 (88%)	3.03 (1.21–6.80)	0.010
**Close contact in South Africa with a person who subsequently tested positive for SARS-CoV-2**					
No/unknown	410 (85%)	19 (5%)	391 (95%)	Ref.	
Yes	72 (15%)	5 (7%)	67 (93%)	1.50 (0.51–3.59)	0.41
**Kept at least a 1.5 m distance**					
Never/rarely	47 (10%)	1 (2%)	46 (98%)	Ref.	
Sometimes	52 (11%)	3 (6%)	49 (94%)	2.48 (0.54–44.0)	0.37
Always/often	379 (79%)	20 (5%)	359 (95%)	2.71 (0.36–53.8)	0.38
**Disinfected hands several times per day**					
Never/rarely	52 (11%)	1 (2%)	51 (98%)	Ref.	
Sometimes	61 (13%)	3 (5%)	58 (95%)	2.56 (0.34–50.9)	0.41
Always/often	366 (76%)	20 (5%)	346 (95%)	2.84 (0.61–50.5)	0.30
**Washed hands several times per day**					
Never/rarely	19 (4%)	2 (11%)	17 (89%)	Ref.	
Sometimes	62 (13%)	1 (2%)	61 (98%)	0.15 (0.01–1.52)	0.12
Always/often	398 (83%)	21 (5%)	377 (95%)	0.50 (0.16–2.99)	0.33
**Wore FFP2 mask**					
Never/rarely	128 (27%)	6 (5%)	122 (95%)	Ref.	
Sometimes	65 (14%)	6 (9%)	59 (91%)	1.97 (0.64–6.07)	0.22
Always/often	281 (59%)	12 (4%)	269 (96%)	0.91 (0.36–2.57)	0.85
**Wore medical mouth-nose mask**					
Never/rarely	192 (44%)	11 (6%)	181 (94%)	Ref.	
Sometimes	58 (13%)	4 (7%)	54 (93%)	1.20 (0.34–3.37)	0.74
Always/often	190 (43%)	9 (5%)	181 (95%)	0.83 (0.34–1.95)	0.66
**Wore fabric mask**					
Never/rarely	367 (86%)	18 (5%)	349 (95%)	Ref.	
Sometimes	16 (4%)	1 (6%)	15 (94%)	1.27 (0.07–5.59)	0.81
Always/often	43 (10%)	5 (12%)	38 (88%)	2.37 (0.82–5.61)	0.072
**Wore mask only indoors**					
Never/rarely	31 (6%)	0 (0%)	31 (100%)	Omitted	
Sometimes	50 (11%)	3 (6%)	47 (94%)	Ref.	
Always/often	392 (83%)	21 (5%)	371 (95%)	0.89 (0.32–3.69)	0.85
**Wore mask indoors and outdoors**					
Never/rarely	250 (54%)	7 (3%)	243 (97%)	Ref.	
Sometimes	107 (23%)	7 (7%)	100 (93%)	2.34 (0.82–6.67)	0.10
Always/often	109 (23%)	10 (9%)	99 (91%)	3.28 (1.29–8.81)	0.013
**Avoided contact with people outside my travel group**					
Never/rarely	165 (35%)	3 (2%)	162 (98%)	Ref.	
Sometimes	150 (31%)	12 (8%)	138 (92%)	4.40 (1.43–19.1)	0.020
Always/often	162 (34%)	9 (6%)	153 (94%)	3.06 (0.93–13.6)	0.089
**Avoided mass transportation**					
Never/rarely	413 (87%)	17 (4%)	396 (96%)	Ref.	
Sometimes	38 (8%)	2 (5%)	36 (95%)	1.26 (0.18–6.20)	0.78
Always/often	26 (5%)	5 (19%)	21 (81%)	4.62 (1.31–17.6)	0.016
**Avoided crowds**					
Never/rarely	194 (41%)	8 (4%)	186 (96%)	Ref	
Sometimes	145 (31%)	11 (8%)	134 (92%)	1.84 (0.76–4.64)	0.18
Always/often	135 (28%)	5 (4%)	130 (96%)	0.90 (0.28–2.63)	0.85

There were missing data for age (n = 11), gender (n = 10), vaccination status (n = 42), previous SARS-CoV-2 infection (n = 26), SARS-CoV-2 test result before departure (n = 61), the time between negative test and departure (n = 70), positive travel partner (n = 52), close contact in South Africa tested positive for SARS-CoV-2 (n = 46), kept distance (n = 50), disinfected hands (n = 49), washed hands (n = 49), wore FFP2-mask (n = 54), wore a medical mouth-nose mask (n = 88), wore fabric mask (n = 102), wore mask only indoors (n = 55), wore mask indoors and outdoors (n = 55), avoiding contact (n = 51), avoiding mass transportation (n = 51), and avoided crowds (n = 54). ^†^ Fully vaccinated was defined as 2 doses of COVID-19 vaccine Comirnaty/Spikevax/Vaxzevria or at least 1 dose of COVID-19 vaccine Janssen with the last shot given at least 14 days ago or recovered from SARS-CoV-2-infection and one dose of any vaccine approved in the EU. Proof of recovery was defined as a previous SARS-CoV-2-infection, which was confirmed by means of a nucleic acid test (e.g., PCR) and the test to prove the prior infection was performed no less than 28 days and no more than 90 days beforehand, according to the German Infection Protection Act.

**Table 4:** Univariable associations of characteristics, prevention measures and in-flight behaviors with self-reported incident infection within 14 days after arrival, among those who tested negative on arrival and had information on incident infection status in the OMTRAIR questionnair.

Characteristics	Total	Tested Positive for SARS-CoV-2 within 14 Days after Arrival		Univariable Analysis	
		Yes	No	RR (95% CI)	*p*-Value
	N = 490 (100%)	n = 21 (4%)	n = 469 (96%)		
**Role in airplane**					
Crew	41 (8.8%)	0 (%)	41 (100%)	Omitted	
Passenger	427 (91%)	20 (5%)	405 (95%)		
**Age, years**					
≤34	113 (23%)	9 (8%)	104 (92%)	Ref.	
≥35	375 (77%)	12 (3%)	363 (97%)	0.40 (0.17–0.96)	0.033
**Gender**					
Male	256 (52%)	5 (2%)	251 (98%)	Ref.	
Female	232 (47%)	16 (7%)	216 (93%)	3.53 (1.41–10.7)	0.012
Diverse	1 (1%)	0 (0%)	1 (100%)	Omitted	
**Vaccination status ^†^**					
Not (fully) vaccinated	78 (17%)	3 (4%)	75 (96%)	Ref.	
Fully vaccinated	382 (83%)	18 (5%)	364 (95%)	1.23 (0.43–5.14)	0.74
**Previous SARS-CoV-2 infection**					
No/unknown	414 (87%)	19 (5%)	395 (95%)	Ref.	
Yes	62 (13%)	1 (2%)	61 (98%)	0.35 (0.02–1.65)	0.304
**Fulfilled entry regulations ^§^**					
No	115 (25%)	1 (1%)	114 (99%)	Ref.	
Yes	338 (75%)	17 (5%)	321 (95%)	5.78 (1.21–104)	0.086
**Type of mask worn during flight**					
Wore FFP2 (KN95) (vs not)	361 (79%)	18 (5%)	343 (95%)	2.37 (0.70–14.7)	0.24
Wore medical mouth-nose mask (vs not)	88 (19%)	2 (2%)	86 (98%)	0.47 (0.075–1.57	0.30
**Wore mask continuously**					
No, not always	401 (88%)	18 (4%)	383 (96%)	Ref.	
Yes, during entire flight	55 (12%)	2 (4%)	53 (96%)	0.81 (0.13–2.70)	0.77
**Consumed food**					
No or once	92 (20%)	4 (4%)	88 (96%)	Ref.	
Several times	361 (80%)	16 (4%)	345 (96%)	1.02 (0.38–3.49)	0.97
**Consumed drinks**					
No or once	30 (7%)	2 (6%)	28 (93%)	Ref.	
Several times	425 (93%)	18 (4%)	407 (96%)	0.64 (0.20–3.88)	0.53
**Visited toilet**					
No or once	96 (21%)	3 (3%)	93 (97%)	Ref.	
Several times	358 (79%)	17 (5%)	341 (95%)	1.52 (0.52–6.41)	0.50
**Conversation with people in close proximity (within 2 m)**					
No or once	235 (52%)	14 (6%)	221 (94%)	Ref.	
Several times	217 (48%)	6 (3%)	211 (97%)	0.46 (0.17–1.13)	0.13
**Travel partner tested positive for SARS-CoV-2**					
No/unknown	399 (88%)	11 (3%)	388 (97%)	Ref.	
Yes	53 (12%)	9 (17%)	44 (83%)	6.16 (2.60–14.2)	<0.001

RR = risk ratio; ^†^ Fully vaccinated was defined as two doses of COVID-19 vaccine Comirnaty/Spikevax/Vaxzevria or at least one dose of COVID-19 vaccine Janssen with the last shot given at least 14 days ago OR recovered from SARS-CoV-2-infection and one dose of any vaccine approved in the EU, according to the German Infection Protection Act. ^§^ Travelers who at any time within the last 10 days prior to entry have visited an area of a variant of concern must complete a digital entry registration before entering Germany, have to provide a negative test result (applies only to persons aged 12 years or older) and have to stay in quarantine for 14 days. This also applies to vaccinated and recovered individuals. There were missing data for the role in the airplane (n = 24), age (n = 2), gender (n = 1), vaccination status (n = 30), previous SARS-CoV-2 infection (n = 14), entry regulations (n = 37), type of mask worn (n = 34), wore mask continuously (n = 45), consumed food (n = 37), consumed drinks (n = 35), visited toilet (n = 36), conversation with people in close proximity (n = 38), and positive travel partner (n = 38).

## Data Availability

Anonymized data are available upon reasonable request.

## Appendix A

**Table A1:** Number and percentage of travelers testing positive on arrival from 19 direct flights from South Africa at Munich Airport, Germany, in November/December 2021.

Date of Flight	Tested for SARS-CoV-2 on Arrival at Munich Airport	Tested Positive for SARS-CoV-2 on Arrival at Munich Airport and Confirmed at the LGL Laboratory
	N = 2728 (100%)	N = 90 (3.3%)
26 November 2021	237 (100%)	2 (0.84%)
28 November 2021	305 (100%)	15 (4.91%)
1 December 2021	262 (100%)	8 (3.05%)
3 December 2021	251 (100%)	11 (4.38%)
4 December 2021	260 (100%)	5 (1.92%)
5 December 2021	203 (100%)	5 (2.46%)
6 December 2021	199 (100%)	5 (2.51%)
8 December 2021	230 (100%)	6 (2.61%)
10 December 2021	155 (100%)	9 (5.81%)
11 December 2021	97 (100%)	4 (4.12%)
12 December 2021	101 (100%)	5 (4.95%)
13 December 2021	48 (100%)	2 (4.17%)
15 December 2021	87 (100%)	3 (3.45%)
17 December 2021	73 (100%)	6 (8.22%)
18 December 2021	52 (100%)	0 (0%)
19 December 2021	54 (100%)	2 (3.70%)
20 December 2021	41 (100%)	2 (4.88%)
22 December 2021	46 (100%)	0 (0%)
23 December 2021	27 (100%)	0 (0%)

**Table A2:** Symptomatology of OMTRAIR participants who tested positive for SARS-CoV-2 Omicron variant at or within 14 days after arrival at Munich Airport, Germany in November/December 2021.

Symptoms	Total
	N = 28 (100%)
Fever (≥38.0 °C/100.4°F)	4 (16%)
Cough	18 (67%)
Rhinitis, sneezing	15 (54%)
Chest pain	3 (12%)
Headache	23 (82%)
Skin rash	1 (4%)
Fatigue or exhaustion	21 (81%)
Chills	8 (32%)
Shortness of breath	8 (32%)
Sore throat	16 (62%)
Decrease or loss of smell or taste	2 (8%)
Pain in limbs	10 (40%)
Diarrhoea	5 (20%)
Nausea, vomiting, abdominal pain	2 (8%)
Conjunctivitis	1 (4%)
Swelling of lymph nodes	3 (12%)
**Hospitalized**	0 (0%)

**Table A3:** Assessment of confounding factors associated with testing positive on arrival in univariable analysis, OMTRAIR study, Germany, November/December 2021.

	Potential Confounders, Added One by One to Each Exposure Model						
Exposures	Age	Gender	Positive Travel Partner	Close Contact	Prevention Measures (Just Sign. Ones)	Previous SARS-CoV-2 Infection (Not Significant)	Time between Test and Departure
**Reporting a positive travel partner**	No change	No change	N/A	No change	Avoided mass transportation: unable to run; Rest: no change	No change	RR decreased by 13% (less sign.) RR = 2.63, 95%CI = 0.97–6.19, *p* = 0.037
**Wore mask indoors and outdoors**	No change	No change	No change	No change	N/A	No change	No change
**Avoided mass transportation**	No change	No change	Unable to run	RR + 11% (more sign.) RR = 5.12, 95%CI = 1.46–19, *p* = 0.010	N/A	No change	RR + 27% RR = 5.86, 95%CI = 1.42–28, *p* = 0.013
**Avoided contact with people outside my travel group**	No change	No change	No change	No change	N/A	No change	RR + 12% (not sign. anymore) RR = 3.88, 95%CI = 0.99–26, *p* = 0.083

**Table A4:** Assessment of confounding factors associated with testing positive within 14 days after arrival (i.e., incident infection) in univariable analysis, OMTRAIR study, Germany, November/December 2021.

	Potential Confounders, Added One by One to Each Exposure Model				
Exposures	Age	Gender	In-Flight Behaviors (Not Evaluated Because All Were Not Associated with the Outcome)	Prevention Measures (Not Evaluated Because All Were Not Associated with the Outcome)	Previous SARS-CoV-2 Infection (Not Significant)
**Age ^1^**	N/A	RR + 20% (not sign. anymore) RR = 0.48, 95%CI = 0.21–1.15, *p* = 0.086	N/A	N/A	No change
**Gender ^1^**	RR—11% (but still sign.) RR = 3.16, 95%CI = 1.25–9.59, *p* = 0.024	N/A	N/A	N/A	No change
**Reporting a positive travel partner**	No change	No change	N/A	N/A	No change

^1^ N.B. No full confounder adjustment is possible, according to the DAG. Nevertheless, we added age and gender to assess whether the associations changed.
